# Blood pressure and dementia risk by physical frailty in the elderly: a nationwide cohort study

**DOI:** 10.1186/s13195-023-01211-y

**Published:** 2023-03-20

**Authors:** Mi Hee Cho, Kyungdo Han, Seungwoo Lee, Su-Min Jeong, Jung Eun Yoo, SangYun Kim, Jinkook Lee, Sohyun Chun, Dong Wook Shin

**Affiliations:** 1 Samsung C&T Medical Clinic, Kangbuk Samsung Hospital, Sungkyunkwan University School of Medicine, Seoul, Republic of Korea; 2 Department of Statistics and Actuarial Science, Soongsil University, Seoul, Republic of Korea; 3 Department of Medical Statistics, College of Medicine, Catholic University of Korea, Seoul, Republic of Korea; 4 Department of Medicine, Seoul National University College of Medicine, Seoul , Republic of Korea; 5 Department of Family Medicine, Health System Gangnam Center, Seoul National University Hospital, Seoul, Republic of Korea; 6 Department of Neurology, Seoul National University Bundang Hospital & Seoul National University College of Medicine, Seongnam, Republic of Korea; 7 Department of Economics & Center for Economic & Social Research, University of Southern California, Los Angeles, CA, USA; 8 International Healthcare Center, Samsung Medical Center, 81 Irwon-Ro, Gangnam-Gu, Seoul, Republic of Korea; 9 Department of Family Medicine/Supportive Care Center, Samsung Medical Center, Sungkyunkwan University School of Medicine, Seoul, Republic of Korea; 10 Department of Clinical Research Design & Evaluation, Samsung Advanced Institute for Health Science & Technology (SAIHST), Sungkyunkwan University, 81 Irwon-Ro, Gangnam-Gu, Seoul, 06351 Republic of Korea

**Keywords:** Dementia, Blood pressure, Timed up and Go, Physical frailty, Elderly population

## Abstract

**Background:**

Midlife hypertension has been recognized as a modifiable risk factor for dementia, but association between blood pressure (BP) in late life and dementia has been inconclusive. In addition, few studies have investigated effects of BP control on dementia incidence in the frail elderly. Thus, this study aimed to investigate the association of BP and dementia incidence with concomitant consideration of physical frailty in the young elderly population.

**Methods:**

Using the Korean National Health Information Database, we identified 804,024 subjects without history of dementia at age 66. Dementia diagnosis was defined with prescription records of anti-dementia drugs and dementia-related diagnostic codes. Physical frailty was measured using the Timed Up and Go test. Association of BP and dementia incidence with concomitant consideration of physical frailty was investigated using Cox hazards analyses.

**Results:**

The risks of Alzheimer’s and vascular dementia increased from systolic BP ≥ 160 and 130–139 mmHg, respectively; a significant association of dementia incidence with low BP was not observed. In the analyses stratified by the physical frailty status, low BP was not associated with increased risks of dementia within the groups both with and without physical frailty.

**Conclusions:**

High BP was associated with increased risks of dementia, especially for vascular dementia, while low BP was not associated with increased risks of any type of dementia in young elderly people, even in those with physical frailty. This study suggests the need for tight BP control in young elderly people, irrespective of frailty status, to prevent dementia and supports the current clinical guidelines of hypertension treatment.

**Supplementary Information:**

The online version contains supplementary material available at 10.1186/s13195-023-01211-y.

## Background

Midlife hypertension has been suggested as a risk factor of dementia in later life, and control of systolic blood pressure (SBP) ≤ 130 mmHg in midlife has been recommended to delay or prevent dementia [1, 2]. However, the association between blood pressure (BP) in late life and dementia development has been inconsistent in previous studies, with the results depending on study settings, such as age of the study population and patterns of BP trajectories [3–7].

Frailty is a common multi-dimensional syndrome that affects physical function (slow walk time, poor grip strength, physical inactivity), metabolism (weight loss), and psychological components (exhaustion) in the elderly population [8]. The association between frailty and dementia risk in the elderly has been reported in previous studies [9–11], and chronic inflammation was suggested as a shared pathogenesis between cognitive impairment and frailty [12].

Beneficial effects of BP control on cardiovascular disease and mortality in the elderly, even with frailty, have been reported [13–15] and are reflected in the recent hypertension guidelines [16, 17]. However, results on the effects of tight BP control on developing dementia in the frail elderly have been inconclusive, and the benefit-risk balance between them according to frailty status, especially in the young old population, was not considered [18, 19].

Thus, the primary objective of this study was to investigate the association of BP and dementia incidence in the young elderly population using a nationwide database. We also aimed to evaluate the association of BP and dementia incidence after stratification by physical frailty, which would provide useful evidence to establish optimal strategies for BP control without increasing dementia risk in the frail elderly.

## Methods

### Data source and study population

This retrospective cohort study was conducted using the National Health Information Database (NHID) provided by the Korean National Health Insurance Service (NHIS) for research purposes. The NHIS provides mandatory universal health insurance to nearly all Koreans (~ 97% of the Korean population) and medical aid to ~ 3% of the population in the lowest income bracket and also manages the relevant administrative processes and information for the beneficiaries. The NHIS provides the National Screening Program for Transitional Ages (NSPTA) to people at age 66, which is regarded as an important transition time to old age, in addition to a free biennial cardiovascular health screening exam to beneficiaries ≥ 40 years or employees at any age [20]. The NSPTA offers screening tests for mental health conditions and geriatric functions as well as tests for cardiovascular health [20]. Therefore, the NHID includes all health information including utilization of medical facilities, such as clinic visits as outpatients, hospitalization, medication prescription, heath examination results and demographic information (age, sex, insurance premium (proxy for income level) and disability status) [21, 22].

The study population included 909,489 subjects who participated in the NSPTA between 2009 and 2012. We excluded subjects with gait disturbance (n = 17,772) due to the possibility of improper measurement of a physical frailty test. Individuals who had missing data for covariates (e.g., smoking status, *n* = 42,485) or who were previously diagnosed with any dementia (*n* = 5,914), end-stage renal disease (*n* = 1,743), or any cancer (*n* = 37,551) before the index date were excluded from the study population. Finally, a total of 804,024 study subjects was included in this study.

### Independent variables: BP and physical function

In the NHIS health exam, a trained clinician measures the brachial BP of participants twice, and the average is recorded. Physical frailty of participants was assessed using the Timed Up and Go (TUG) test, which is a validated tool to assess the functional mobility of the elderly and to predict the patient's ability to go outside alone safely [23]. The standardized protocol of the TUG test is to measure the time required for the participant to get up from a chair, walk 3 m along a line drawn on the floor at a comfortable pace, turn around, return to the chair, and sit down on the chair. A TUG test result ≥ 10 s is defined as gait impairment [23].

### Outcome: dementia incidence

The outcome of this study was newly diagnosed dementia. Dementia-related codes of the International Classification of Diseases, 10^th^ Revision (ICD-10) and prescription data for anti-dementia drugs were used to identify cases of dementia. Patients with newly diagnosed dementia were defined as those with prescription records for anti-dementia drugs at least twice with diagnostic codes of Alzheimer’s disease (AD, ICD-10 F00 or G30), vascular dementia (VaD, ICD-10 F01), or other dementia (ICD-10 F02, F03, G23.1 or G31). The follow-up started on the date of the NSPTA and ended at the date of dementia diagnosis (incidence), death from any other cause, or December 31, 2017, whichever came first.

### Measurements and definitions of co-variates

In the NHIS screening examination, anthropometric examinations (height, weight, waist circumference, and BP), laboratory tests (fasting glucose, lipid profile, hemoglobin, and creatinine) and questionnaires on health behaviors (smoking status, alcohol consumption, and exercise) are included for cardiovascular risk screening. The smoking status was divided into 3 categories: non-smoker, ex-smoker, and current smoker. Drinking habit was also grouped into 3 categories: none (0 g/day), mild (< 30 g/day), and heavy (≥ 30 g/day). Regular exercise was defined as moderate to vigorous intensity of physical activity ≥ 3 days per week. Body mass index (BMI) was calculated by dividing participant weight by the square of participant height (kg/m^2^). Categorization of income level was based on monthly insurance premiums, as insurance premiums are imposed according to income level in Korea.

The NSPTA for particpants at age 66 also includes a the Geriatric Depression Scale (GDS) [24] and the Prescreening Korean Dementia Screening Questionnaire (KDSQ-P) for cognitive impairment [25]. Presence of depression was defined if participants answered ‘yes’ to any of the three selected items on the GDS: loss of activity or interest, feelings of worthlessness, and feelings of hopelessness. The KDSQ-P consists of 5 items, scored 0–2 (with a total score range 0–10) [25], and participants with a score ≥ 4 undergo further tests for diagnosis of dementia.

Administration of antihypertensive medications was defined as the presence of at least one claimed prescription record of antihypertensive agents per year along with a diagnostic code of hypertension (ICD-10 codes I10-11). Diabetes was defined as either the presence of at least one claimed diagnostic codes under ICD–10 codes E11–14 with prescription of antidiabetic medication or a fasting glucose level ≥ 126 mg/dL. Chronic kidney disease (CKD) was defined as a glomerular filtration rate (GFR) < 60 ml/min/1.73 m^2^, as estimated by the Modification of Diet in Renal Disease (MDRD) equation. Other comorbidities were defined by diagnostic codes only: ICD-10 codes J40-47 for chronic obstructive pulmonary disease (COPD), ICD-10 codes I20-25 for ischemic heart disease (IHD), and ICD-10 codes I63 or I64 for stroke.

### Statistical analysis

Descriptive statistics using independent t-tests for continuous variables and chi-square tests for categorical variables were used to summarize the baseline characteristics of the study population according to the dementia incidence. Incidence rates (IR) for all dementia, AD and VaD were expressed as cases per 1000 person-years.

Cox proportional hazards analyses were performed to estimate hazard ratios (HRs) with 95% confidence interval (CI) of dementia and its subtypes according to BP and functional status measured by the TUG test. In this study, BP was categorized as follows: SBP < 100, 100–109, 110–119, 120–129, 130–139, 140–149, 150–159, and ≥ 160 mmHg, and DBP < 60, 60–69, 70–79, 80–89, 90–99, 100–109, and ≥ 110 mmHg. For analysis purposes, SBP 110–119 mmHg and DBP 70–79 mmHg were considered as the reference BP for SBP and DBP, respectively. To conduct stratified analyses according to functional status, participants were categorized into 3 groups, based on results of the TUG test: Group 1 (< 10 s), Group 2 (10–14.9 s), and Group 3 (≥ 15 s).

Cox analyses were performed in two steps. First, we assessed the associations of dementia incidence with SBP and DBP in the total study population. All models were adjusted for the following variables: sex, income level, smoking status, alcohol consumption, exercise, BMI, hemoglobin, KDSQ scores, administration of antihypertensive medication, and relevant comorbid conditions. As a previous study reported the association of anemia with dementia incidence [15, 26], hemoglobin also was included as a covariate. Second, after stratified into 3 categories based on TUG score, Group 1 (< 10 s), Group 2 (10–14.9 s), and Group 3 (≥ 15 s), the same Cox analyses were performed in each group as follows: 1) Model 1 was analyzed using the group with reference BP and the TUG score < 10 s as a reference for all categories of the TUG score to investigate risks of dementia incidence with concomitant consideration of both BP and physical frailty, and 2) Model 2 was analyzed using the group with reference BP as a reference within each category of the TUG score to study an independent effect of BP on dementia incidence within a group with different levels of physical frailty. Statistical analyses were conducted using SAS version 9.4 (SAS Institute Inc., Cary, NC, USA), and two-tailed *P*-values of < 0.05 were considered to indicate statistical significance.

## Results

### Baseline characteristics

A total of study population was 804,024. All participants were 66 years old at baseline and the mean follow-up duration was 6.6 years (range 5–9 years). The baseline characteristics of the study population are presented in Table 1. During follow-up duration, 42,476 individuals (IR = 7.95 per 1000 person-year) were identified as newly diagnosed with dementia. Among the newly diagnosed dementia cases, 31,612 cases were diagnosed with AD (IR = 5.91 per 1000 person-year) and 5,655 were diagnosed with VaD (IR = 1.06 per 1000 person-year). Only the mean values of SBP and DBP were not statistically different between the non-dementia and dementia groups (Table 1).

**Table 1:** Baseline characteristics of the study population according to future dementia incidence

	**Total**	**Future dementia incidence**		***p*** ** value**
		**No**	**Yes**	
All individuals, n (%)^a^	804,024	761,548 (94.7)	42,476 (5.3)	
Age, years^b^	66	66	66	
Sex, n (%)^a^				< 0.001
Male	367,898 (45.8)	351,442 (46.2)	16,456 (38.7)	
Female	436,126 (54.2)	410,106 (53.9)	26,020(61.3)	
Income, n (%)^a^				< 0.001
Medicaid (lowest)	28,334 (3.5)	24,735 (3.2)	3,599 (8.5)	
Quartile 1	197,920 (24.6)	187,784 (24.7)	10,136 (23.9)	
Quartile 2	154,420 (19.2)	146,332 (19.2)	8,088 (19.0)	
Quartile 3	216,033 (26.9)	204,934 (26.9)	11,099 (26.1)	
Quartile 4 (highest)	207,317 (25.8)	197,763 (26.0)	9,554 (22.5)	
Smoking, n (%)^a^				< 0.001
Never	561,805 (69.9)	530,628 (69.7)	31,177 (73.4)	
Former	134,344 (16.7)	129,161 (17.0)	5,183 (12.2)	
Current	107,875 (13.4)	101,759 (13.4)	6,116 (14.4)	
Drink, n (%)^a^				< 0.001
Non	574,077 (71.4)	541,513 (71.1)	32,564 (76.7)	
Mild	196,939 (24.5)	188,801 (24.8)	8,138 (19.2)	
Heavy	33,008 (4.1)	31,234 (4.1)	1,774 (4.2)	
Regular exercise, n (%)	373,084 (46.4)	356.325 (46.8)	16,759 (39.5)	< 0.001
Hypertension, n (%)	353,947 (44.0)	333,437 (43.8)	20,510 (48.3)	< 0.001
DM, n (%)	159,302 (19.8)	147,836 (19.4)	11,466 (27.0)	< 0.001
CKD by GFR (mL/min/1.73 m^2^), n (%)^a^				< 0.001
GFR ≥ 60	707,082 (87.9)	671,203 (88.1)	35,879 (84.5)	
GFR < 60	90,550 (11.3)	84,447 (11.1)	6,103 (14.4)	
GFR < 30	6,392 (0.8)	5,898 (0.8)	494 (1.2)	
COPD, n (%)	105,557 (13.1)	99,225 (13.0)	6,332 (14.9)	< 0.001
IHD, n (%)	97,475 (12.1)	90,800 (11.9)	6,675 (15.7)	< 0.001
Stroke, n (%)	34,142 (4.2)	29,762 (3.9)	4,380 (10.3)	< 0.001
Depression, n (%)	162,767 (20.2)	150,368 (19.7)	12,399 (29.2)	< 0.001
KDSQ score, mean (SD)	0.5 (2.3)	1.3 (2.5)	2.0 (3.4)	< 0.001
TUG results, second, n (%)^a^				< 0.001
< 10	583,900 (72.6)	555,539 (72.9)	28,361 (66.8)	
10–14.9	191,883 (23.9)	179,953 (23.6)	11,930 (28.1)	
≥ 15	28,241 (3.5)	26,056 (3.4)	2,185 (5.1)	
BMI, kg/m^2^, mean (SD)	24.3 (3.0)	24.3 (3.0)	24.2 (3.2)	< 0.001
SBP, mmHg, mean (SD)	128.7 (15.4)	128.7 (15.4)	128.7 (16.2)	0.375
DBP, mmHg, mean (SD)	77.6 (10.0)	78.0 (9.8)	78.0 (10.1)	0.409
Hemoglobin, g/dl, mean (SD)	13.6 (1.4)	13.6 (1.4)	13.4 (1.4)	< 0.001
FBG, mg/dl, mean (SD)	103.6 (25.9)	103.3 (25.5)	107.2 (32.7)	< 0.001
TC, mg/dl, mean (SD)	198.0 (38.5)	198.0 (38.4)	197.4 (40.3)	0.002

*DM* diabetes mellitus, *CKD* chronic kidney disease, *GFR* glomerular filtration rate, *COPD* chronic obstructive pulmonary disease, *IHD* ischemic heart disease, *KDSQ* Korean Dementia Screening Questionnaire, *BMI* body mass index, *TUG* timed up and go, *SBP* systolic blood pressure, *DBP* diastolic blood pressure, *FBG* fasting blood glucose, *TC* total cholesterol

^a^ Column percentage

^b^ Since this study included participants of the NSPTA, all participants were 66 years old at baseline

### Dementia risk according to BP in all participants

**Fig. 1:**
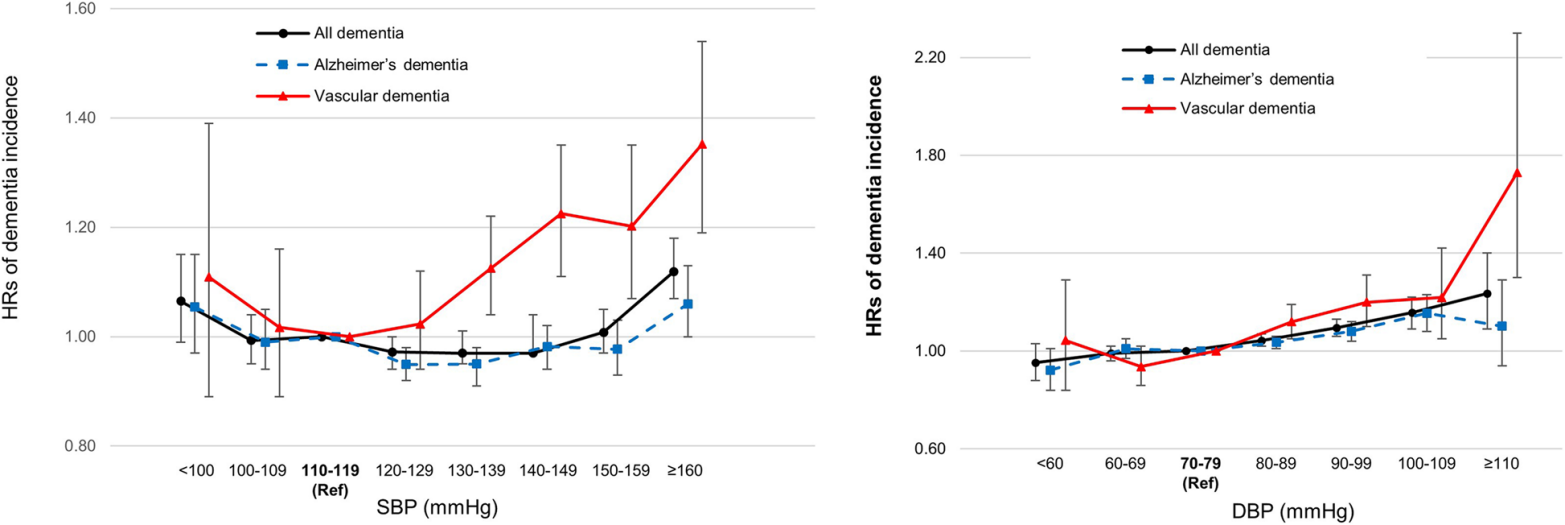
Hazard ratios and 95% confidence intervals of dementia incidence according to BP in the total study population. Reference groups are those with reference BP (SBP 110–119 mmHg and DBP 70–79 mmHg). Adjusted for sex, income, smoking status, alcohol consumption, exercise, BMI, hemoglobin, KDSQ score, administration of antihypertensive medication, DM, CKD, COPD, IHD, stroke, and depression

The risks of any dementia and AD significantly increased from SBP ≥ 160 mmHg (adjusted HR [aHR] 1.12, 95% CI 1.07–1.18 for all dementia and aHR 1.06, 95% CI 1.00–1.13 for AD) and from DBP 80–89 mmHg (aHR 1.04, 95% CI 1.02–1.07 for all dementia and aHR 1.04, 95% CI 1.01–1.06 for AD), compared with the group with reference BP (SBP 110–119 mmHg or DBP 70–79 mmHg). The risks of VaD began to increase from SBP 130–139 mmHg (aHR 1.13, 95% CI 1.04–1.22) and from DBP 80–89 mmHg (aHR 1.12, 95% CI 1.05–1.19). No elevated risk of dementia was observed for SBP and DBP lower than the reference BP (Fig. 1, Supplementary Table S1).

### Dementia risk according to the BP level and the TUG results

**Fig. 2:**
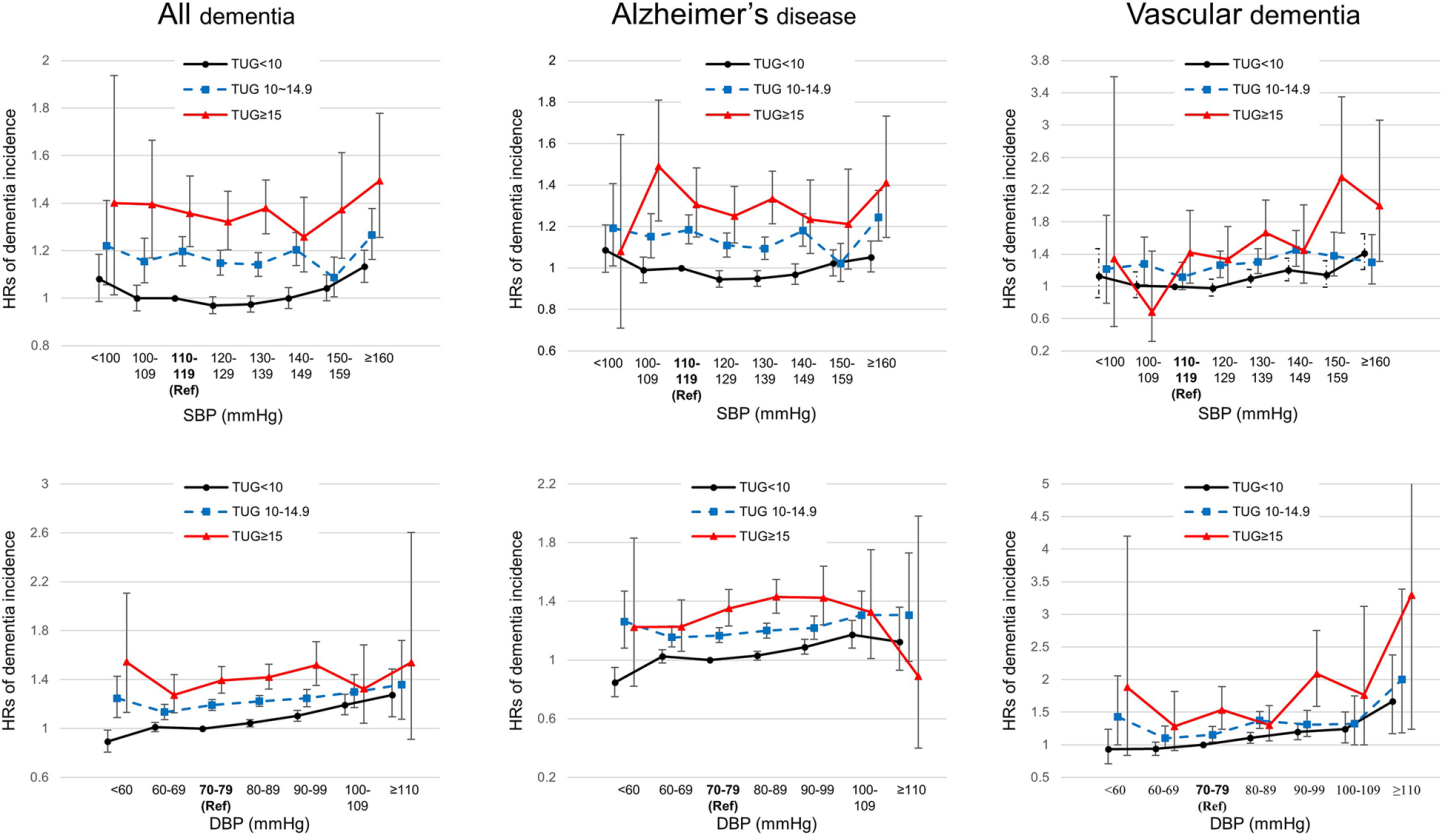
Hazard ratios and 95% confidence intervals of dementia incidence according to BP using the group with reference BP and TUG result < 10 s as a reference for all categories of the TUG score. The reference SBP and DBP were 110–119 and 70–79 mmHg, respectively. The risks of dementia incidence were evaluated with concomitant consideration of both BP and physical frailty. Adjusted for sex, income, smoking status, alcohol consumption, exercise, BMI, hemoglobin, KDSQ score, administration of antihypertensive medication, DM, CKD, COPD, IHD, stroke, and depression

When using the group with SBP 110–119 mmHg and the TUG result < 10 s as a reference, the risks of all dementia and VaD began to increase from SBP ≥ 160 mmHg (aHR 1.13, 95% CI 1.07–1.20) and SBP 140–149 mmHg (aHR 1.20, 95% CI 1.07–1.35) in the normal TUG group (TUG < 10 s), while any increased risk of AD was not observed in this group (Fig. 2, Supplementary Table S2). No elevated risk of dementia was observed for patients with SBP lower than the reference SBP (110–110 mmHg) in the normal TUG group. In contrast, the risk of all kinds of dementia incidences increased at almost all SBP levels in groups with impaired performance on the TUG test (TUG 10–14.9 and TUG ≥ 15 s).

When using the group with DBP 70–79 mmHg and the TUG result < 10 s as a reference, the risks of dementia increased from DBP ≥ 80 mmHg for all dementia (aHR 1.04, 95% CI 1.01–1.07), AD (aHR 1.03, 95% CI 1.00–1.06), and VaD (aHR 1.10, 95% CI 1.02–1.19) in the normal TUG group (Fig. 2, Supplementary Table S3). DBP levels lower than the reference DBP were not associated with any increases of dementia risks in this group. However, in the groups with TUG ≥ 10 s, risks for all kinds of dementia increased at almost all DBP levels.

### Dementia risks by BP within the group stratified by TUG results

**Fig. 3:**
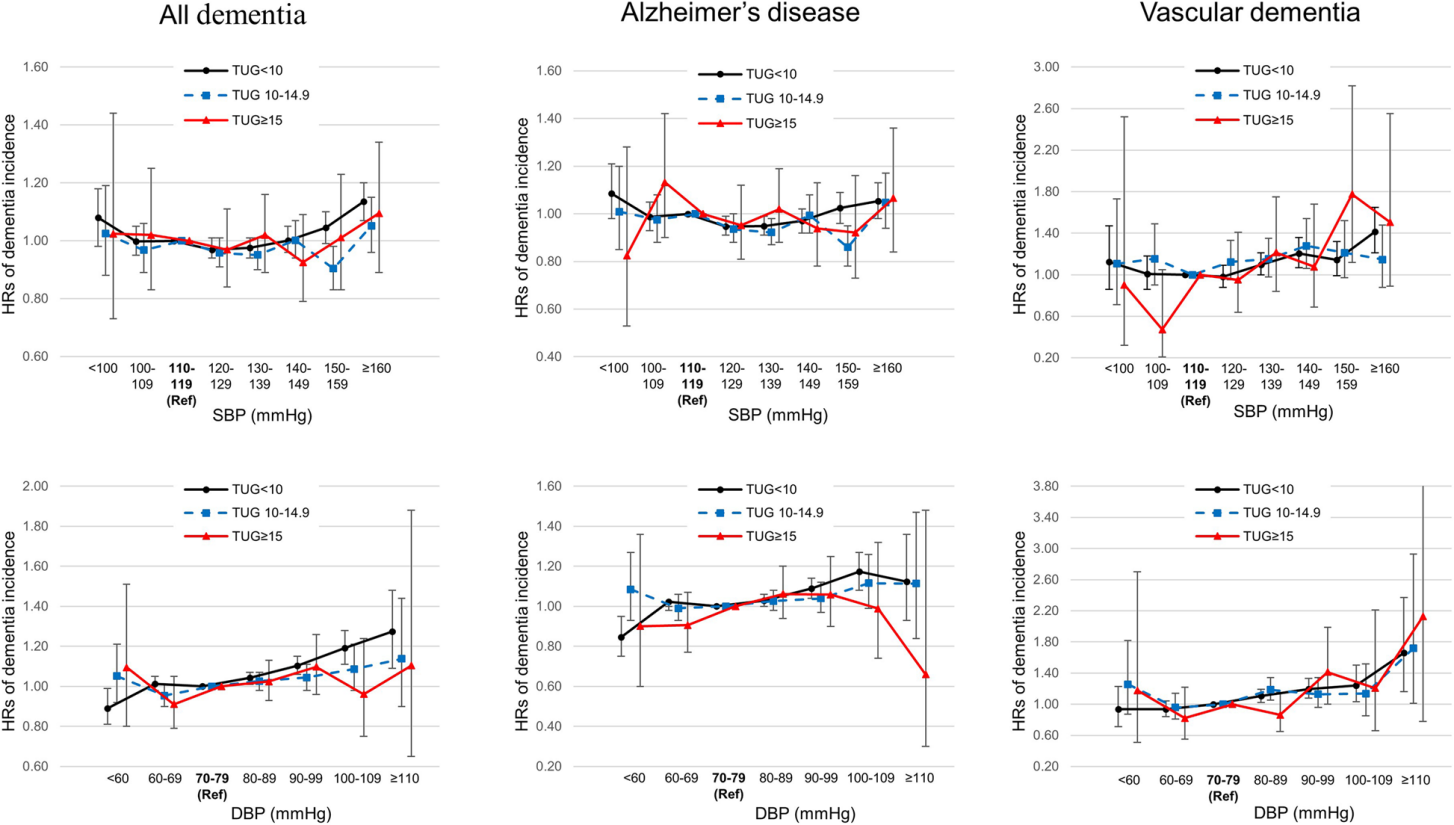
Hazard ratios and 95% confidence intervals of dementia incidence according to BP using the group with SBP 110–119 mmHg and DBP 70–79 mmHg as a reference within each category of TUG performance. The independent effect of BP on dementia incidence was evaluated within a group with different levels of physical frailty. Adjusted for sex, income, smoking status, alcohol consumption, exercise, BMI, hemoglobin, KDSQ score, administration of antihypertensive medication, DM, CKD, COPD, IHD, stroke, and depression

In the abnormal TUG groups (TUG 10–14.9 and TUG ≥ 15 s), increased risks of all dementia and AD risks were not observed at all SBP levels, while the risk of VaD increased from SBP ≥ 140–150 mmHg (aHR 1.28, 95% CI 1.06–1.54 in the TUG 10–14.9 group and aHR 1.78, 95% CI 1.12–2.82 in the TUG ≥ 15 group), compared with the group with reference SBP (Fig. 3, Supplementary Table S4). Similar patterns were observed in analyses with DBP (Fig. 3, Supplementary Table S5). Elevated risks of VaD were observed with DPB 80–90 mmHg (aHR 1.19, 95% CI 1.05–1.34) in the TUG 10–14.9 group and with DBP 90–99 mmHg (aHR 1.41, 95% CI 1.00–1.99) in the TUG ≥ 15 group, compared with the group with reference DBP (Fig. 3, Supplementary Table S5).

### Subgroup analyses stratified by status of hypertension treatment

In subgroup analyses stratified by administration of antihypertensive medications, the associations between BP and dementia showed similar tendencies in both subgroups, but to a different degree (Supplementary Table S6 and S7). In the group with TUG ≥ 15 s, the risks of dementia, especially AD, increased higher with increasing BP in individuals without antihypertensive medication compared with those with the medication.

## Discussion

In this study, we investigated the association between BP and dementia risk with concomitant consideration of physical frailty in the young elderly population; this aim represents a novelty of this study. Other study strengths further include the following: 1) the use of a nationwide database with a population at a homogenous age and a wide-range of medical information to eliminate potential bias; 2) the use of both dementia-related disease codes and prescription records of anti-dementia drugs to define newly diagnosed dementia to minimize overestimation of dementia cases; and 3) the evaluation of physical frailty using the TUG test, which is an easy-to-use examination in outpatient clinic.

Midlife hypertension is a well-established risk factor of dementia in late life. However, the risks of late-life hypertension for dementia incidence have been inconsistent in previous researches [3, 4, 7, 27]. One study even reported that individuals managing SBP ≥ 130 mmHg with antihypertensive treatment, compared with those with SBP < 130 mmHg, showed less cognitive decline in participants aged ≥ 75 years [28]. However, in the current study, increased risks of both AD and VaD were observed at high BP (SBP ≥ 160 for AD, SBP ≥ 130 mmHg for VaD, and DBP ≥ 80 mmHg for all dementia) in the total study population, and similar patterns were found in subgroup analysis stratified by status of antihypertensive treatment. This finding provides robust evidence to support the need for BP control, at least in the young elderly, to lower the risks of dementia incidence. The association between high BP and dementia might be explained by the shared pathophysiology of vascular risk factors. Hypertension can cause atherosclerosis in both small and large vessels, which is a predisposing condition of dementia [4, 29]. Additionally, a recent review suggested that hypertension might cause reduction in clearance of harmful proteins such as β-amyloid [6].

The risks of dementia and cognitive decline with low BP in the elderly have been investigated, but the results were inconclusive [4, 7, 27, 30]. Cerebral hypo-perfusion, resulting in ischemic insults, was a commonly suggested mechanism of the increased dementia risk with low BP [31, 32]. However, recent studies reported no evidence of a harmful effect of lowering BP with antihypertensive medication on cerebral blood flow even in older adults [33, 34] or of impairment of cerebral autoregulation in AD [35], which supports our finding that low BP was not associated with increased risks of dementia in young elderly people. The Atherosclerosis Risk in Communities (ARIC) study reported an increased risk of dementia in the midlife hypertension and late-life hypotension group in the elderly population (mean age (standard deviation [SD]), 75 (5) years) [7].The Framingham Offspring study also reported that a steep decline in SBP during mid- to late life was associated with an increased dementia risk, while low SBP (< 100 mmHg) and DBP (< 70 mmHg) in late life (mean age (SD), 69 (6) years) were not [4]. The recent report from the Systolic Blood Pressure Intervention Trial–Memory and Cognition in Decreased Hypertension (SPINT MIND) also found no increased risks of dementia or cognitive decline in the group with intensive SBP control (SBP < 120 mmHg) [19], which was consistent with our findings. Some possible reasons for these inconsistent findings are that the risk of low BP for dementia incidence might depend on age or trajectories of BP and that the causality between late-life declines in BP and dementia incidence might not be clear [7].

The association of dementia and cognitive decline with frailty has been reported in several previous studies [9–11, 36]. A shared basic pathogenesis of cognitive impairment and frailty is chronic inflammation [12]. In addition, pro-inflammatory cytokines and genetic variants have been reported as related to both cognitive impairment and physical frailty [12]. Evaluation of physical frailty in the elderly population is complex. A study using UK Biobank reported an association between individual component of frailty and dementia incidence and found that slow gait speed increased the risk of dementia incidence (HR 1.41, 95% CI 1.10–1.79) [10], suggesting that functional immobility is an independent risk of dementia. Instead of full assessment of frailty, other previous studies used a relatively simple physical function test to evaluate frailty and reported that gait speed was an independent risk factor of dementia [10, 37].

Previous research also showed that physical frailty measured by the TUG test was associated with dementia incidence and cognitive decline [38, 39], which was consistent with the results of this study. In this study, physical frailty measured by the TUG test, regardless of BP, was associated with increased risks of dementia compared with the group with the reference BP and without physical frailty. This finding suggests that physical frailty may have a stronger association with dementia incidence than elevated BP or alter the magnitude of the effect of BP on dementia incidence. One neuropathology study reported that the degree of frailty might alter the likelihood to be expressed as clinical dementia in individuals with AD pathology; the severe frailty, the higher likelihood to be expressed as AD even with same degree of AD pathology [40].

A notable finding of this study is that low BP was not associated with increased risks of all types of dementia in early elderly people even with physical frailty as it was in people without physical frailty. This finding suggests that BP control in the early elderly with physical frailty seems to be beneficial in lowering the risk of dementia and that even intensive BP lowering may not increase dementia risks, which may be in line with the recent hypertension guideline where advanced old age and frailty were removed from considerations for caution to decide intensive hypertension therapy [17, 41]. However, a limited number of studies have investigated the composite effects of frailty and BP on dementia risks, and further research is required to confirm our findings.

The subgroup analyses of this study found that irrespective of status of antihypertensive treatment, low BP was not associated with an increased risk of dementia in the total study population of the early elderly, whereas high BP tended to increase the risks of dementia, especially VaD. These results suggest that screening and treatment of hypertension would be important not only in middle-aged patients but also in the early elderly to prevent dementia, supporting the recent report from the SPINT MIND [19]. The SPINT MIND study reported that compared with standard BP control, intensive BP control in the elderly with hypertension (mean age (SD), 67.9 (9.4) years) reduced the risks of incident mild cognitive impairment (MCI) and a combined outcome of incident MCI and dementia [19].

This study has several limitations. First, BP was measured only at baseline, and a chronological change of BP was not considered in this study. Second, caution should be applied in generalizing our findings, especially regarding the TUG test, to all ages in the elderly population, since this study was conducted only with a relatively young elderly population. Third, since frailty is composed of multi-dimensional features including physical, metabolic, and psychological components, the TUG test is a narrow measure to assess frailty in the elderly population. Further research using frailty index assessed with various variables is needed to clarify the effect of frailty on the association between blood pressure and dementia incidence. Finally, considering the time lag until the onset of dementia, the follow-up duration in this study may not be long enough to reveal clear associations among BP, physical frailty and dementia.

## Conclusion

In conclusion, in this nationwide population-based cohort study, we demonstrated that high BP was associated with increased risks of dementia, especially for VaD, while low BP was not associated with increased risks of all types of dementia in young elderly people, even in those with physical frailty. Our study suggests the need for tight BP control in young elderly people, irrespective of their frailty status, to prevent dementia and supports the current clinical guidelines of hypertension treatment. Further studies, particularly well-designed clinical trials, are warranted to examine optimizing BP level to reduce dementia risks.

## Supplementary Information

**Additional file 1:Supplementary Table S1.** Hazard ratios and incidence rates of dementia according to systolic blood pressure compared with the group with reference BP (SBP 110–119 mmHg and DBP 70–79 mmHg). **Supplementary Table S2.** Hazard ratios and incidence rates of dementia according to systolic blood pressure using the group with SBP 110–119 mmHg and TUG result < 10 s as a reference. **Supplementary Table S3**. Hazard ratios and incidence rates of dementia according to diastolic blood pressure using the group with DBP 70–79 mmHg and TUG result < 10 s as a reference. **Supplementary Table S4.** Hazard ratios and incidence rates of dementia according to systolic blood pressure using the group with SBP 110–119 mmHg as a reference within each category of TUG result. **Supplementary Table S5.** Hazard ratios and incidence rates of dementia according to DBP using the group with DBP 70–79 mmHg as a reference within each category of TUG result. **Supplementary Table S6.** Stratified analyses by hypertensive status: hazard ratios and incidence rates of dementia according to SBP using the group with SBP 110–119 mmHg and the TUG result < 10 s as a reference. **Supplementary Table S7.** Stratified analyses by hypertensive status: hazard ratios and incidence rates of dementia according to diastolic blood pressure when using the group with DBP 70–79 mmHg and TUG result < 10 s as a reference.

## Data Availability

The datasets used and/or analyzed during the current study are available from the corresponding author on reasonable request.

## Abbreviations

AD: Alzheimer’s disease
ARIC: Atherosclerosis Risk in Communities study
BMI: Body mass index
BP: Blood pressure
CKD: Chronic kidney disease
COPD: Chronic obstructive pulmonary disease
DBP: Diastolic blood pressure
GDS: Geriatric Depression Scale
IHD: Ischemic heart disease
KDSQ-P: Prescreening Korean Dementia Screening Questionnaire
MCI: Mild cognitive impairment
MDRD: Modification of Diet in Renal Disease equation
NHID: National Health Information Database
NHIS: National Health Insurance Service
NSPTA: National Screening Program for Transitional Ages
SBP: Systolic blood pressure
SPINT MIND: Systolic Blood Pressure Intervention Trial – Memory and Cognition in Decreased Hypertension
TUG: Timed Up and Go test
VaD: Vascular dementia
