# Supplementary material for: Blood pressure and dementia risk by physical frailty in the elderly: a nationwide cohort study
Source: Alzheimers Res Ther. 2023 Mar 20;15:56. doi: 10.1186/s13195-023-01211-y (PMC10026431; doi:10.1186/s13195-023-01211-y)
Supplement: Supplementary file 1 — Additional file 1:Supplementary Table S1. Hazard ratios and incidence rates of dementia according to systolic blood pressure compared with the group with reference BP (SBP 110–119 mmHg and DBP 70–79 mmHg). Supplementary Table S2. Hazard ratios and incidence rates of dementia according to systolic blood pressure using the group with SBP 110–119 mmHg and TUG result < 10 s as a reference. Supplementary Table S3. Hazard ratios and incidence rates of dementia according to diastolic blood pressure using the group with DBP 70–79 mmHg and TUG result < 10 s as a reference. Supplementary Table S4. Hazard ratios and incidence rates of dementia according to systolic blood pressure using the group with SBP 110–119 mmHg as a reference within each category of TUG result. Supplementary Table S5. Hazard ratios and incidence rates of dementia according to DBP using the group with DBP 70–79 mmHg as a reference within each category of TUG result. Supplementary Table S6. Stratified analyses by hypertensive status: hazard ratios and incidence rates of dementia according to SBP using the group with SBP 110–119 mmHg and the TUG result < 10 s as a reference. Supplementary Table S7. Stratified analyses by hypertensive status: hazard ratios and incidence rates of dementia according to diastolic blood pressure when using the group with DBP 70–79 mmHg and TUG result < 10 s as a reference. [file 13195_2023_1211_MOESM1_ESM.docx]

**Supplementary Table S1. Hazard ratios and incidence rates of dementia according to systolic blood pressure compared with the group with reference BP (SBP 110–119 mmHg and DBP 70–79 mmHg)**

|  |  |  | All dementia | | | | | Alzheimer’s disease | | | | | Vascular dementia | | | | | |
| --- | --- | --- | --- | --- | --- | --- | --- | --- | --- | --- | --- | --- | --- | --- | --- | --- | --- | --- |
|  | BP | N | N | Person-years | IR | HR* | (95% CI) | N | Person-years | IR | HR* | (95% CI) | N | Person-years | IR | HR* | (95% CI) |  |
| SBP | <100 | 11,771 | 721 | 76307 | 9.45 | 1.07 | (0.99–1.15) | 550 | 76307 | 7.21 | 1.05 | (0.97–1.15) | 83 | 76307 | 1.09 | 1.11 | (0.89–1.39) |  |
|  | 100–109 | 45,677 | 2,502 | 300831 | 8.32 | 0.99 | (0.95–1.04) | 1,921 | 300831 | 6.39 | 0.99 | (0.94–1.05) | 290 | 300831 | 0.96 | 1.02 | (0.89–1.16) |  |
|  | 110–119 | 142,349 | 7,639 | 945853 | 8.08 | 1.00 | (reference) | 5,845 | 945853 | 6.18 | 1.00 | (reference) | 885 | 945853 | 0.94 | 1.00 | (reference) |  |
|  | 120–129 | 189,884 | 9,671 | 1257824 | 7.69 | 0.97 | (0.94–1.00) | 7,185 | 1257824 | 5.71 | 0.95 | (0.92–0.98) | 1,208 | 1257824 | 0.96 | 1.02 | (0.94–1.12) |  |
|  | 130–139 | 232,924 | 11,922 | 1554894 | 7.67 | 0.98 | (0.95–1.01) | 8,795 | 1554894 | 5.66 | 0.95 | (0.91–0.98) | 1,667 | 1554894 | 1.07 | **1.13** | **(1.04–1.22)** |  |
|  | 140–149 | 953,81 | 5,061 | 632875 | 8.00 | 1.01 | (0.97–1.04) | 3,728 | 632875 | 5.89 | 0.98 | (0.94–1.02) | 770 | 632875 | 1.22 | **1.23** | **(1.11–1.35)** |  |
|  | 150–159 | 522,24 | 2,836 | 349714 | 8.11 | 1.01 | (0.97–1.05) | 2,070 | 349714 | 5.92 | 0.98 | (0.93–1.03) | 426 | 349714 | 1.22 | **1.20** | **(1.07–1.35)** |  |
|  | ≥160 | 338,14 | 2,124 | 227328 | 9.34 | **1.12** | **(1.07–1.18)** | 1,518 | 227328 | 6.68 | **1.06** | **(1.00–1.13)** | 326 | 227328 | 1.43 | **1.35** | **(1.19–1.54)** |  |
| DBP | <60 | 12,252 | 659 | 79882 | 8.25 | 0.95 | (0.88–1.03) | 480 | 79882 | 6.01 | 0.92 | (0.84–1.01) | 88 | 79882 | 1.10 | 1.04 | (0.84–1.29) |  |
|  | 60–69 | 107,537 | 5,742 | 708022 | 8.11 | 0.99 | (0.96–1.02) | 4,393 | 708022 | 6.20 | 1.01 | (0.97–1.05) | 672 | 708022 | 0.95 | 0.94 | (0.86–1.02) |  |
|  | 70–79 | 273,497 | 14,092 | 1814871 | 7.76 | 1.00 | (reference) | 10,548 | 1814871 | 5.81 | 1.00 | (reference) | 1,780 | 1814871 | 0.98 | 1.00 | (reference) |  |
|  | 80–89 | 296,441 | 15,526 | 1978195 | 7.85 | **1.04** | **(1.02–1.07)** | 11,482 | 1978195 | 5.80 | **1.04** | **(1.01–1.06)** | 2,162 | 1978195 | 1.09 | **1.12** | **(1.05–1.19)** |  |
|  | 90–99 | 88,391 | 4,902 | 590551 | 8.30 | **1.09** | **(1.06–1.13)** | 3,583 | 590551 | 6.07 | **1.08** | **(1.04–1.12)** | 718 | 590551 | 1.22 | **1.20** | **(1.10–1.31)** |  |
|  | 100–109 | 22,030 | 1,303 | 148151 | 8.80 | **1.16** | **(1.09–1.22)** | 961 | 148151 | 6.49 | **1.15** | **(1.08–1.23)** | 186 | 148151 | 1.26 | **1.22** | **(1.05–1.42)** |  |
|  | ≥110 | 3,876 | 252 | 25955 | 9.71 | **1.24** | **(1.09–1.40)** | 165 | 25955 | 6.36 | 1.10 | (0.94–1.29) | 49 | 25955 | 1.89 | **1.73** | **(1.30–2.30)** |  |

BMI, body mass index; BP, blood pressure; CI, confidence interval; CKD, chronic kidney disease; COPD, chronic obstructive pulmonary disease; DBP, diastolic blood pressure; DM, diabetes mellitus; HR, hazard ratio; IHD, ischemic heart disease; IR, incidence rate (per 1000); KDSQ, Korean Dementia Screening Questionnaire; N, number; SBP, systolic blood pressure

* Adjusted for sex, income, smoking status, alcohol consumption, exercise, BMI, hemoglobin, KDSQ score, administration of antihypertensive medication, DM, CKD, COPD, IHD, stroke, and depression.

**Supplementary Table S2. Hazard ratios and incidence rates of dementia according to systolic blood pressure using the group with SBP 110–119 mmHg and TUG result < 10 sec as a reference.**

|  |  |  | All dementia | | | | | Alzheimer’s disease | | | | | Vascular dementia | | | | |
| --- | --- | --- | --- | --- | --- | --- | --- | --- | --- | --- | --- | --- | --- | --- | --- | --- | --- |
| TUG | SBP | N | N | Person-years | IR | HR* | (95% CI) | N | Person-years | IR | HR* | (95% CI) | N | Person-years | IR | HR* | (95% CI) |
| <10 | <100 | 8,641 | 494 | 56081 | 8.81 | 1.08 | (0.99–1.19) | 384 | 56081 | 6.85 | 1.09 | (0.98–1.21) | 58 | 56081 | 1.03 | 1.13 | (0.86–1.47) |
|  | 100–109 | 33,805 | 1,719 | 222526 | 7.72 | 1.00 | (0.95–1.06) | 1,311 | 222526 | 5.89 | 0.99 | (0.93–1.05) | 200 | 222526 | 0.90 | 1.01 | (0.86–1.18) |
|  | 110–119 | 106,412 | 5,261 | 706314 | 7.45 | 1.00 | (reference) | 4,039 | 706314 | 5.72 | 1.00 | (reference) | 621 | 706314 | 0.88 | 1.00 | (reference) |
|  | 120–129 | 136,885 | 6,379 | 905333 | 7.05 | 0.97 | (0.93–1.01) | 4,757 | 905333 | 5.25 | **0.95** | **(0.91–0.99)** | 779 | 905333 | 0.86 | 0.98 | (0.88–1.09) |
|  | 130–139 | 168,735 | 7,893 | 1123313 | 7.03 | 0.98 | (0.94–1.01) | 5,841 | 1123313 | 5.20 | **0.95** | **(0.91–0.99)** | 1,099 | 1123313 | 0.98 | 1.10 | (0.99–1.21) |
|  | 140–149 | 68,041 | 3,300 | 451063 | 7.32 | 1.00 | (0.96–1.05) | 2,418 | 451063 | 5.36 | 0.97 | (0.92–1.02) | 505 | 451063 | 1.12 | **1.20** | **(1.07–1.35)** |
|  | 150–159 | 37,476 | 1,928 | 250543 | 7.70 | 1.04 | (0.99–1.10) | 1,429 | 250543 | 5.70 | 1.02 | (0.96–1.09) | 271 | 250543 | 1.08 | 1.14 | (0.99–1.32) |
|  | ≥160 | 23,905 | 1,387 | 160660 | 8.63 | **1.13** | **(1.07–1.20)** | 973 | 160660 | 6.06 | 1.05 | (0.98–1.13) | 224 | 160660 | 1.39 | **1.41** | **(1.21–1.65)** |
| 10-14.9 | <100 | 2,709 | 190 | 17578 | 10.81 | **1.22** | **(1.06–1.41)** | 144 | 17578 | 8.19 | **1.19** | **(1.01–1.41)** | 21 | 17578 | 1.19 | 1.22 | (0.79–1.88) |
|  | 100–109 | 10,351 | 657 | 68339 | 9.61 | **1.16** | **(1.07–1.25)** | 506 | 68339 | 7.40 | **1.15** | **(1.05–1.26)** | 83 | 68339 | 1.21 | **1.28** | **(1.02–1.61)** |
|  | 110–119 | 31,585 | 2,036 | 210678 | 9.66 | **1.20** | **(1.14–1.26)** | 1,553 | 210678 | 7.37 | **1.18** | **(1.12–1.26)** | 222 | 210678 | 1.05 | 1.12 | (0.96–1.30) |
|  | 120–129 | 46,430 | 2,805 | 308766 | 9.08 | **1.15** | **(1.10–1.20)** | 2,075 | 308766 | 6.72 | **1.11** | **(1.05–1.17)** | 370 | 308766 | 1.20 | **1.26** | **(1.11–1.44)** |
|  | 130–139 | 55,913 | 3,380 | 375992 | 8.99 | **1.14** | **(1.09–1.19)** | 2,474 | 375992 | 6.58 | **1.09** | **(1.04–1.15)** | 473 | 375992 | 1.26 | **1.30** | **(1.16–1.47)** |
|  | 140–149 | 23,613 | 1,498 | 156968 | 9.54 | **1.20** | **(1.14–1.28)** | 1,114 | 156968 | 7.10 | **1.18** | **(1.10–1.26)** | 227 | 156968 | 1.45 | **1.45** | **(1.25–1.69)** |
|  | 150–159 | 12,777 | 757 | 85979 | 8.80 | **1.09** | **(1.01–1.17)** | 540 | 85979 | 6.28 | 1.02 | (0.93–1.12) | 122 | 85979 | 1.42 | **1.38** | **(1.13–1.67)** |
|  | ≥160 | 8,505 | 607 | 57220 | 10.61 | **1.27** | **(1.16–1.38)** | 452 | 57220 | 7.90 | **1.25** | **(1.13–1.37)** | 80 | 57220 | 1.40 | **1.30** | **(1.03–1.64)** |
| ≥15 | <100 | 421 | 37 | 2649 | 13.97 | **1.40** | **(1.01–1.94)** | 22 | 2649 | 8.31 | 1.08 | (0.71–1.64) | 4 | 2649 | 1.51 | 1.35 | (0.50–3.60) |
|  | 100–109 | 1,521 | 126 | 9967 | 12.64 | **1.40** | **(1.17–1.67)** | 104 | 9967 | 10.43 | **1.49** | **(1.23–1.81)** | 7 | 9967 | 0.70 | 0.68 | (0.32–1.44) |
|  | 110–119 | 4,352 | 342 | 28861 | 11.85 | **1.36** | **(1.22–1.51)** | 253 | 28861 | 8.77 | **1.31** | **(1.15–1.48)** | 42 | 28861 | 1.46 | **1.42** | **(1.04–1.94)** |
|  | 120–129 | 6,569 | 487 | 43726 | 11.14 | **1.32** | **(1.20–1.45)** | 353 | 43726 | 8.07 | **1.25** | **(1.12–1.39)** | 59 | 43726 | 1.35 | **1.34** | **(1.02–1.74)** |
|  | 130–139 | 8,276 | 649 | 55589 | 11.68 | **1.38** | **(1.27–1.50)** | 480 | 55589 | 8.63 | **1.33** | **(1.21–1.47)** | 95 | 55589 | 1.71 | **1.67** | **(1.34–2.07)** |
|  | 140–149 | 3,727 | 263 | 24845 | 10.59 | **1.26** | **(1.11–1.42)** | 196 | 24845 | 7.89 | **1.23** | **(1.07–1.42)** | 38 | 24845 | 1.53 | **1.45** | **(1.04–2.01)** |
|  | 150–159 | 1,971 | 151 | 13192 | 11.45 | **1.37** | **(1.17–1.61)** | 101 | 13192 | 7.66 | 1.21 | (0.99–1.48) | 33 | 13192 | 2.50 | **2.36** | **(1.66–3.35)** |
|  | ≥160 | 1,404 | 130 | 9448 | 13.76 | **1.49** | **(1.26–1.78)** | 93 | 9448 | 9.84 | **1.41** | **(1.15–1.73)** | 22 | 9448 | 2.33 | **2.00** | **(1.31–3.06)** |

BMI, body mass index; CI, confidence interval; CKD, chronic kidney disease; COPD, chronic obstructive pulmonary disease; DM, diabetes mellitus; HR, hazard ratio; IHD, ischemic heart disease; IR, incidence rate (per 1000); KDSQ, Korean Dementia Screening Questionnaire; N, number; SBP, systolic blood pressure; TUG, timed up and go

* Adjusted for sex, income, smoking status, alcohol consumption, exercise, BMI, hemoglobin, KDSQ score, administration of antihypertensive medication, DM, CKD, COPD, IHD, stroke, and depression.

**Supplementary Table S3. Hazard ratios and incidence rates of dementia according to diastolic blood pressure using the group with DBP 70–79 mmHg and TUG result < 10 sec as a reference.**

|  |  |  | All dementia | | | | | Alzheimer’s disease | | | | | Vascular dementia | | | | |
| --- | --- | --- | --- | --- | --- | --- | --- | --- | --- | --- | --- | --- | --- | --- | --- | --- | --- |
| TUG | DBP | N | N | Person-years | IR | HR* | (95% CI) | N | Person-years | IR | HR* | (95% CI) | N | Person-years | IR | HR* | (95% CI) |
| <10 | <60 | 8,758 | 404 | 57210 | 7.06 | 0.89 | (0.81–0.99) | 291 | 57210 | 5.09 | 0.85 | (0.75–0.95) | 52 | 57210 | 0.91 | 0.94 | (0.71–1.24) |
|  | 60–69 | 79,022 | 3,975 | 520252 | 7.64 | 1.01 | (0.98–1.05) | 3,042 | 520252 | 5.85 | 1.02 | (0.98–1.07) | 458 | 520252 | 0.88 | 0.94 | (0.84–1.04) |
|  | 70–79 | 202,052 | 9,544 | 1339428 | 7.13 | 1.00 | (reference) | 7,186 | 1339428 | 5.36 | 1.00 | (reference) | 1,214 | 1339428 | 0.91 | 1.00 | (reference) |
|  | 80–89 | 213,150 | 10,199 | 1418582 | 7.19 | **1.04** | **(1.01–1.07)** | 7,537 | 1418582 | 5.31 | **1.03** | **(1.00–1.06)** | 1411 | 1418582 | 0.99 | **1.10** | **(1.02–1.19)** |
|  | 90–99 | 62,665 | 3,209 | 418074 | 7.68 | **1.10** | **(1.06–1.15)** | 2,357 | 418074 | 5.64 | **1.09** | **(1.04–1.14)** | 468 | 418074 | 1.12 | **1.20** | **(1.08–1.33)** |
|  | 100–109 | 15,526 | 862 | 104046 | 8.28 | **1.19** | **(1.11–1.28)** | 630 | 104046 | 6.06 | **1.17** | **(1.08–1.27)** | 123 | 104046 | 1.18 | **1.24** | **(1.03–1.50)** |
|  | ≥110 | 2,727 | 168 | 18241 | 9.21 | **1.28** | **(1.10–1.49)** | 109 | 18241 | 5.98 | 1.12 | (0.93–1.36) | 31 | 18241 | 1.70 | **1.67** | **(1.17–2.38)** |
| 10-14.9 | <60 | 3,037 | 215 | 19830 | 10.84 | **1.25** | **(1.09–1.43)** | 165 | 19830 | 8.32 | **1.26** | **(1.08–1.47)** | 30 | 19830 | 1.51 | **1.43** | **(1.00–2.06)** |
|  | 60–69 | 24,881 | 1,501 | 163909 | 9.16 | **1.13** | **(1.07–1.20)** | 1,157 | 163909 | 7.06 | **1.15** | **(1.09–1.23)** | 181 | 163909 | 1.10 | 1.10 | (0.94–1.29) |
|  | 70–79 | 62,703 | 3,868 | 417404 | 9.27 | **1.19** | **(1.15–1.24)** | 2,864 | 417404 | 6.86 | **1.17** | **(1.12–1.22)** | 472 | 417404 | 1.13 | **1.15** | **(1.04–1.28)** |
|  | 80–89 | 72,506 | 4,500 | 487225 | 9.24 | **1.22** | **(1.18–1.27)** | 3,319 | 487225 | 6.81 | **1.20** | **(1.15–1.25)** | 653 | 487225 | 1.34 | **1.37** | **(1.25–1.51)** |
|  | 90–99 | 22,198 | 1,403 | 148749 | 9.43 | **1.25** | **(1.18–1.32)** | 1,023 | 148749 | 6.88 | **1.22** | **(1.14–1.30)** | 197 | 148749 | 1.32 | **1.31** | **(1.13–1.53)** |
|  | 100–109 | 5,573 | 373 | 37781 | 9.87 | **1.30** | **(1.17–1.44)** | 280 | 37781 | 7.41 | **1.31** | **(1.16–1.47)** | 51 | 37781 | 1.35 | **1.32** | **(1.00–1.75)** |
|  | ≥110 | 985 | 70 | 6621 | 10.57 | **1.36** | **(1.08–1.72)** | 50 | 6621 | 7.55 | 1.31 | (0.99–1.73) | 14 | 6621 | 2.11 | **2.00** | **(1.18–3.39)** |
| ≥15 | <60 | 457 | 40 | 2842 | 14.07 | **1.54** | **(1.13–2.11)** | 24 | 2842 | 8.44 | 1.22 | (0.82–1.83) | 6 | 2842 | 2.11 | 1.88 | (0.84–4.20) |
|  | 60–69 | 3,634 | 266 | 23861 | 11.15 | **1.27** | **(1.13–1.44)** | 194 | 23861 | 8.13 | **1.23** | **(1.06–1.41)** | 33 | 23861 | 1.38 | 1.28 | (0.91–1.82) |
|  | 70–79 | 8,742 | 680 | 58039 | 11.72 | **1.39** | **(1.29–1.51)** | 498 | 58039 | 8.58 | **1.35** | **(1.23–1.48)** | 94 | 58039 | 1.62 | **1.53** | **(1.24–1.89)** |
|  | 80–89 | 10,785 | 827 | 72388 | 11.42 | **1.42** | **(1.32–1.53)** | 626 | 72388 | 8.65 | **1.43** | **(1.32–1.55)** | 98 | 72388 | 1.35 | **1.30** | **(1.06–1.60)** |
|  | 90–99 | 3,528 | 290 | 23728 | 12.22 | **1.52** | **(1.35–1.71)** | 203 | 23728 | 8.56 | **1.42** | **(1.24–1.64)** | 53 | 23728 | 2.23 | **2.09** | **(1.59–2.75)** |
|  | 100–109 | 931 | 68 | 6324 | 10.75 | **1.33** | **(1.04–1.68)** | 51 | 6324 | 8.06 | **1.33** | **(1.01–1.75)** | 12 | 6324 | 1.90 | **1.76** | **(1.00–3.12)** |
|  | ≥110 | 164 | 14 | 1093 | 12.80 | 1.54 | (0.91–2.60) | 6 | 1093 | 5.49 | 0.89 | (0.40–1.98) | 4 | 1093 | 3.66 | **3.30** | **(1.24–8.79)** |

BMI, body mass index; CI, confidence interval; CKD, chronic kidney disease; COPD, chronic obstructive pulmonary disease; DBP, diastolic blood pressure; DM, diabetes mellitus; HR, hazard ratio; IHD, ischemic heart disease; IR, incidence rate (per 1000); KDSQ, Korean Dementia Screening Questionnaire; N, number; TUG, timed up and go

* Adjusted for sex, income, smoking status, alcohol consumption, exercise, BMI, hemoglobin, KDSQ score, administration of antihypertensive medication, DM, CKD, COPD, IHD, stroke, and depression.

**Supplementary Table S4. Hazard ratios and incidence rates of dementia according to systolic blood pressure using the group with SBP 110–119 mmHg as a reference within each category of TUG result.**

|  |  |  | All dementia | | | | | Alzheimer disease | | | | | Vascular dementia | | | | |
| --- | --- | --- | --- | --- | --- | --- | --- | --- | --- | --- | --- | --- | --- | --- | --- | --- | --- |
| TUG | SBP | N | N | Person-years | IR | HR* | (95% CI) | N | Person-years | IR | HR* | (95% CI) | N | Person-years | IR | HR* | (95% CI) |
| <10 | <100 | 8,641 | 494 | 56081 | 8.81 | 1.08 | (0.98–1.18) | 384 | 56081 | 6.85 | 1.09 | (0.98–1.21) | 58 | 56081 | 1.03 | 1.12 | (0.86–1.47) |
|  | 100-109 | 33,805 | 1719 | 222526 | 7.72 | 1.00 | (0.95–1.05) | 1,311 | 222526 | 5.89 | 0.99 | (0.93–1.05) | 200 | 222526 | 0.90 | 1.01 | (0.86–1.18) |
|  | 110-119 | 106,412 | 5261 | 706314 | 7.45 | 1.00 | (reference) | 4,039 | 706314 | 5.72 | 1.00 | (reference) | 621 | 706314 | 0.88 | 1.00 | (reference) |
|  | 120-129 | 136,885 | 6379 | 905333 | 7.05 | 0.97 | (0.94–1.01) | 4,757 | 905333 | 5.25 | **0.95** | **(0.91–0.99)** | 779 | 905333 | 0.86 | 0.98 | (0.88–1.09) |
|  | 130-139 | 168,735 | 7893 | 1123313 | 7.03 | 0.98 | (0.94–1.01) | 5,841 | 1123313 | 5.20 | **0.95** | **(0.91–0.99)** | 1,099 | 1123313 | 0.98 | **1.10** | **(1.00–1.21)** |
|  | 140-149 | 68041 | 3300 | 451063 | 7.32 | 1.00 | (0.96–1.05) | 2,418 | 451063 | 5.36 | 0.97 | (0.92–1.02) | 505 | 451063 | 1.12 | **1.20** | **(1.07–1.36)** |
|  | 150-159 | 37476 | 1928 | 250543 | 7.70 | 1.05 | (0.99–1.10) | 1,429 | 250543 | 5.70 | 1.02 | (0.96–1.09) | 271 | 250543 | 1.08 | 1.15 | (0.99–1.32) |
|  | ≥160 | 23905 | 1387 | 160660 | 8.63 | **1.14** | **(1.07–1.20)** | 973 | 160660 | 6.06 | 1.05 | (0.98–1.13) | 224 | 160660 | 1.39 | **1.42** | **(1.21–1.65)** |
| 10–14.9 | <100 | 2,709 | 190 | 17578 | 10.81 | 1.03 | (0.88–1.19) | 144 | 17578 | 8.19 | 1.01 | (0.85–1.20) | 21 | 17578 | 1.19 | 1.11 | (0.71–1.73) |
|  | 100-109 | 10,351 | 657 | 68339 | 9.61 | 0.97 | (0.89–1.06) | 506 | 68339 | 7.40 | 0.98 | (0.88–1.08) | 83 | 68339 | 1.21 | 1.16 | (0.90–1.49) |
|  | 110-119 | 31,585 | 2036 | 210678 | 9.66 | 1.00 | (reference) | 1,553 | 210678 | 7.37 | 1.00 | (reference) | 222 | 210678 | 1.05 | 1.00 | (reference) |
|  | 120-129 | 46,430 | 2805 | 308766 | 9.08 | 0.96 | (0.91–1.01) | 2,075 | 308766 | 6.72 | **0.94** | **(0.88–1.00)** | 370 | 308766 | 1.20 | 1.12 | (0.95–1.33) |
|  | 130-139 | 55,913 | 3380 | 375992 | 8.99 | 0.95 | (0.90–1.01) | 2,474 | 375992 | 6.58 | **0.92** | **(0.87–0.98)** | 473 | 375992 | 1.26 | 1.15 | (0.98–1.35) |
|  | 140-149 | 23,613 | 1498 | 156968 | 9.54 | 1.00 | (0.94–1.07) | 1,114 | 156968 | 7.10 | 0.99 | (0.92–1.08) | 227 | 156968 | 1.45 | **1.28** | **(1.06–1.54)** |
|  | 150-159 | 12,777 | 757 | 85979 | 8.80 | **0.90** | **(0.83–0.98)** | 540 | 85979 | 6.28 | **0.86** | **(0.78–0.95)** | 122 | 85979 | 1.42 | 1.21 | (0.97–1.52) |
|  | ≥160 | 8,505 | 607 | 57220 | 10.61 | 1.05 | (0.96–1.15) | 452 | 57220 | 7.90 | 1.05 | (0.94–1.17) | 80 | 57220 | 1.40 | 1.15 | (0.88–1.48) |
| ≥15 | <100 | 421 | 37 | 2649 | 13.97 | 1.02 | (0.73–1.44) | 22 | 2649 | 8.31 | 0.83 | (0.53–1.28) | 4 | 2649 | 1.51 | 0.90 | (0.32–2.52) |
|  | 100-109 | 1,521 | 126 | 9967 | 12.64 | 1.02 | (0.83–1.25) | 104 | 9967 | 10.43 | 1.13 | (0.90–1.42) | 7 | 9967 | 0.70 | 0.47 | (0.21–1.05) |
|  | 110-119 | 4,352 | 342 | 28861 | 11.85 | 1.00 | (reference) | 253 | 28861 | 8.77 | 1.00 | (reference) | 42 | 28861 | 1.46 | 1.00 | (reference) |
|  | 120-129 | 6,569 | 487 | 43726 | 11.14 | 0.97 | (0.84–1.11) | 353 | 43726 | 8.07 | 0.95 | (0.81–1.12) | 59 | 43726 | 1.35 | 0.95 | (0.64–1.41) |
|  | 130-139 | 8,276 | 649 | 55589 | 11.68 | 1.02 | (0.89–1.16) | 480 | 55589 | 8.63 | 1.02 | (0.88–1.19) | 95 | 55589 | 1.71 | 1.21 | (0.84–1.75) |
|  | 140-149 | 3,727 | 263 | 24845 | 10.59 | 0.93 | (0.79–1.09) | 196 | 24845 | 7.89 | 0.94 | (0.78–1.13) | 38 | 24845 | 1.53 | 1.08 | (0.69–1.68) |
|  | 150-159 | 1,971 | 151 | 13192 | 11.45 | 1.01 | (0.83–1.23) | 101 | 13192 | 7.66 | 0.92 | (0.73–1.16) | 33 | 13192 | 2.50 | **1.78** | **(1.12–2.82)** |
|  | ≥160 | 1404 | 130 | 9448 | 13.76 | 1.10 | (0.89–1.34) | 93 | 9448 | 9.84 | 1.07 | (0.84–1.36) | 22 | 9448 | 2.33 | 1.51 | (0.89–2.55) |

BMI, body mass index; CI, confidence interval; CKD, chronic kidney disease; COPD, chronic obstructive pulmonary disease; DM, diabetes mellitus; HR, hazard ratio; IHD, ischemic heart disease; IR, incidence rate (per 1000); KDSQ, Korean Dementia Screening Questionnaire; N, number; SBP, systolic blood pressure; TUG, timed up and go

* Adjusted for sex, income, smoking status, alcohol consumption, exercise, BMI, hemoglobin, KDSQ score, administration of antihypertensive medication, DM, CKD, COPD, IHD, stroke, and depression.

**Supplementary Table S5. Hazard ratios and incidence rates of dementia according to DBP using the group with DBP 70–79 mmHg as a reference within each category of TUG result.**

|  |  |  | All dementia | | | | | Alzheimer’s disease | | | | | Vascular dementia | | | | |
| --- | --- | --- | --- | --- | --- | --- | --- | --- | --- | --- | --- | --- | --- | --- | --- | --- | --- |
| TUG | DBP | N | N | Person-years | IR | HR* | (95% CI) | N | Person-years | IR | HR* | (95% CI) | N | Person-years | IR | HR* | (95% CI) |
| <10 | <60 | 8,758 | 404 | 57210 | 7.06 | **0.89** | **(0.81–0.99)** | 291 | 57210 | 5.09 | **0.85** | **(0.75–0.95)** | 52 | 57210 | 0.91 | 0.93 | (0.71–1.23) |
|  | 60–69 | 79,022 | 3,975 | 520252 | 7.64 | 1.01 | (0.98–1.05) | 3,042 | 520252 | 5.85 | 1.02 | (0.98–1.07) | 458 | 520252 | 0.88 | 0.94 | (0.84–1.04) |
|  | 70–79 | 202,052 | 9,544 | 1339428 | 7.13 | 1.00 | (reference) | 7,186 | 1339428 | 5.36 | 1.00 | (reference) | 1214 | 1339428 | 0.91 | 1.00 | (reference) |
|  | 80–89 | 213,150 | 10,199 | 1418582 | 7.19 | **1.04** | **(1.01–1.07)** | 7,537 | 1418582 | 5.31 | **1.03** | **(1.00–1.06)** | 1411 | 1418582 | 0.99 | **1.11** | **(1.02–1.19)** |
|  | 90–99 | 62,665 | 3,209 | 418074 | 7.68 | **1.10** | **(1.06–1.15)** | 2,357 | 418074 | 5.64 | **1.09** | **(1.04–1.14)** | 468 | 418074 | 1.12 | **1.20** | **(1.08–1.33)** |
|  | 100–109 | 15,526 | 862 | 104046 | 8.28 | **1.19** | **(1.11–1.28)** | 630 | 104046 | 6.06 | **1.17** | **(1.08–1.27)** | 123 | 104046 | 1.18 | **1.24** | **(1.03–1.50)** |
|  | ≥110 | 2,727 | 168 | 18241 | 9.21 | **1.27** | **(1.09–1.48)** | 109 | 18241 | 5.98 | 1.12 | (0.93–1.36) | 31 | 18241 | 1.70 | **1.66** | **(1.16–2.37)** |
| 10–14.9 | <60 | 3,037 | 215 | 19830 | 10.84 | 1.05 | (0.92–1.21) | 165 | 19830 | 8.32 | 1.09 | (0.93–1.27) | 30 | 19830 | 1.51 | 1.26 | (0.87–1.82) |
|  | 60–69 | 24,881 | 1,501 | 163909 | 9.16 | 0.95 | (0.90–1.01) | 1,157 | 163909 | 7.06 | 0.99 | (0.93–1.06) | 181 | 163909 | 1.10 | 0.96 | (0.81–1.14) |
|  | 70–79 | 62,703 | 3,868 | 417404 | 9.27 | 1.00 | (reference) | 2,864 | 417404 | 6.86 | 1.00 | (reference) | 472 | 417404 | 1.13 | 1.00 | (reference) |
|  | 80–89 | 72,506 | 4,500 | 487225 | 9.24 | 1.03 | (0.98–1.07) | 3,319 | 487225 | 6.81 | 1.03 | (0.98–1.08) | 653 | 487225 | 1.34 | **1.19** | **(1.05–1.34)** |
|  | 90–99 | 22,198 | 1,403 | 148749 | 9.43 | 1.05 | (0.98–1.11) | 1,023 | 148749 | 6.88 | 1.04 | (0.97–1.12) | 197 | 148749 | 1.32 | 1.13 | (0.96–1.34) |
|  | 100–109 | 5,573 | 373 | 37781 | 9.87 | 1.09 | (0.98–1.21) | 280 | 37781 | 7.41 | 1.12 | (0.99–1.26) | 51 | 37781 | 1.35 | 1.14 | (0.85–1.52) |
|  | ≥110 | 985 | 70 | 6621 | 10.57 | 1.14 | (0.90–1.44) | 50 | 6621 | 7.55 | 1.11 | (0.84–1.47) | 14 | 6621 | 2.11 | **1.72** | **(1.01–2.93)** |
| ≥15 | <60 | 457 | 40 | 2842 | 14.07 | 1.10 | (0.80–1.51) | 24 | 2842 | 8.44 | 0.90 | (0.60–1.36) | 6 | 2842 | 2.11 | 1.18 | (0.51–2.70) |
|  | 60–69 | 3,634 | 266 | 23861 | 11.15 | 0.91 | (0.79–1.05) | 194 | 23861 | 8.13 | 0.91 | (0.77–1.07) | 33 | 23861 | 1.38 | 0.82 | (0.55–1.22) |
|  | 70–79 | 8,742 | 680 | 58039 | 11.72 | 1.00 | (reference) | 498 | 58039 | 8.58 | 1.00 | (reference) | 94 | 58039 | 1.62 | 1.00 | (reference) |
|  | 80–89 | 10,785 | 827 | 72388 | 11.42 | 1.02 | (0.93–1.13) | 626 | 72388 | 8.65 | 1.06 | (0.94–1.20) | 98 | 72388 | 1.35 | 0.86 | (0.65–1.15) |
|  | 90–99 | 3,528 | 290 | 23728 | 12.22 | 1.10 | (0.96–1.26) | 203 | 23728 | 8.56 | 1.06 | (0.90–1.25) | 53 | 23728 | 2.23 | **1.41** | **(1.00–1.99)** |
|  | 100–109 | 931 | 68 | 6324 | 10.75 | 0.96 | (0.75–1.24) | 51 | 6324 | 8.06 | 0.99 | (0.74–1.32) | 12 | 6324 | 1.90 | 1.21 | (0.66–2.21) |
|  | ≥110 | 164 | 14 | 1093 | 12.80 | 1.10 | (0.65–1.88) | 6 | 1093 | 5.49 | 0.66 | (0.30–1.48) | 4 | 1093 | 3.66 | 2.13 | (0.78–5.81) |

BMI, body mass index; CI, confidence interval; CKD, chronic kidney disease; COPD, chronic obstructive pulmonary disease; DBP, diastolic blood pressure; DM, diabetes mellitus; HR, hazard ratio; IHD, ischemic heart disease; IR, incidence rate (per 1000); KDSQ, Korean Dementia Screening Questionnaire; N, number; TUG, timed up and go

* Adjusted for sex, income, smoking status, alcohol consumption, exercise, BMI, hemoglobin, KDSQ score, administration of antihypertensive medication, DM, CKD, COPD, IHD, stroke, and depression.

**Supplementary Table S6. Stratified analyses by hypertensive status: hazard ratios and incidence rates of dementia according to SBP using the group with SBP 110–119 mmHg and the TUG result < 10 sec as a reference.**

|  |  | Without antihypertensive medication | | | | | | | | | With antihypertensive medication | | | | | | | | |
| --- | --- | --- | --- | --- | --- | --- | --- | --- | --- | --- | --- | --- | --- | --- | --- | --- | --- | --- | --- |
|  |  | All dementia | | | Alzheimer’s disease | | | Vascular dementia | | | All dementia | | | Alzheimer’s disease | | | Vascular dementia | | |
| TUG | SBP | N | HR* | (95% CI) | N | HR* | (95% CI) | N | HR* | (95% CI) | N | HR* | (95% CI) | N | HR* | (95% CI) | N | HR* | (95% CI) |
| Total | <100 | 538 | 1.04 | (0.95–1.14) | 419 | 1.04 | (0.94–1.16) | 54 | 1.02 | (0.77–1.35) | 183 | 1.15 | (0.99–1.34) | 131 | 1.10 | (0.92–1.31) | 29 | 1.36 | (0.93–1.98) |
|  | 100–109 | 1,713 | 0.96 | (0.91–1.01) | 1,340 | 0.97 | (0.91–1.03) | 179 | 0.96 | (0.81–1.14) | 789 | 1.08 | (1.00–1.17) | 581 | 1.06 | (0.96–1.16) | 111 | 1.14 | (0.92–1.41) |
|  | 110–119 | 4,882 | 1.00 | (reference) | 3,769 | 1.00 | (reference) | 521 | 1.00 | (reference) | 2,757 | 1.00 | (reference) | 2,076 | 1.00 | (reference) | 364 | 1.00 | (reference) |
|  | 120–129 | 5,421 | 0.97 | (0.93–1.01) | 4,129 | **0.96** | **(0.92–1.00)** | 588 | 0.97 | (0.87–1.10) | 4,250 | 0.97 | (0.93–1.02) | 3,056 | **0.93** | **(0.88–0.98)** | 620 | 1.07 | (0.94–1.22) |
|  | 130–139 | 5,874 | 0.98 | (0.94–1.01) | 4,405 | **0.95** | **(0.91–1.00)** | 743 | 1.12 | (0.99–1.25) | 6,048 | 0.98 | (0.93–1.02) | 4,390 | **0.94** | **(0.89–0.99)** | 924 | **1.13** | **(1.00–1.27)** |
|  | 140–149 | 1,953 | 1.06 | (1.00–1.11) | 1,470 | 1.04 | (0.98–1.10) | 277 | **1.34** | **(1.16–1.56)** | 3,108 | 0.97 | (0.92–1.02) | 2,258 | **0.94** | **(0.88–1.00)** | 493 | **1.16** | **(1.02–1.33)** |
|  | 150–159 | 961 | 1.04 | (0.97–1.11) | 714 | 1.01 | (0.93–1.09) | 137 | **1.31** | **(1.08–1.58)** | 1,875 | 0.99 | (0.93–1.05) | 1,356 | 0.95 | (0.89–1.02) | 289 | 1.15 | (0.99–1.35) |
|  | ≥160 | 624 | **1.13** | **(1.04–1.23)** | 458 | 1.08 | (0.98–1.19) | 92 | **1.46** | **(1.17–1.83)** | 1,500 | **1.11** | **(1.04–1.18)** | 1,060 | 1.04 | (0.96–1.12) | 234 | **1.31** | **(1.11–1.55)** |
| <10 | <100 | 375 | 1.07 | (0.96–1.19) | 295 | 1.09 | (0.97–1.23) | 40 | 1.10 | (0.79–1.53) | 119 | 1.13 | (0.94–1.36) | 89 | 1.11 | (0.90–1.38) | 18 | 1.23 | (0.77–1.99) |
|  | 100–109 | 1,208 | 0.99 | (0.93–1.06) | 937 | 1.00 | (0.92–1.07) | 127 | 0.98 | (0.80–1.20) | 511 | 1.03 | (0.93–1.13) | 374 | 0.98 | (0.88–1.10) | 73 | 1.07 | (0.83–1.39) |
|  | 110–119 | 3,367 | 1.00 | (reference) | 2,589 | 1.00 | (reference) | 363 | 1.00 | (reference) | 1,894 | 1.00 | (0.93–1.13) | 1,450 | 1.00 | (reference) | 258 | 1.00 | (reference) |
|  | 120–129 | 3,617 | 0.98 | (0.93–1.02) | 2,770 | 0.97 | (0.92–1.03) | 381 | 0.94 | (0.81–1.08) | 2,762 | 0.96 | (0.90–1.01) | 1,987 | **0.90** | **(0.84–0.96)** | 398 | 1.01 | (0.86–1.18) |
|  | 130–139 | 3,986 | 0.99 | (0.95–1.04) | 3,018 | 0.98 | (0.93–1.04) | 487 | 1.09 | (0.95–1.25) | 3,907 | 0.95 | (0.90–1.00) | 2,823 | **0.90** | **(0.84–0.96)** | 612 | 1.09 | (0.94–1.26) |
|  | 140–149 | 1,268 | 1.05 | (0.98–1.12) | 944 | 1.02 | (0.95–1.10) | 187 | **1.37** | **(1.15–1.64)** | 2,032 | 0.96 | (0.90–1.02) | 1,474 | **0.91** | **(0.85–0.98)** | 318 | 1.10 | (0.93–1.29) |
|  | 150–159 | 666 | 1.08 | (0.99–1.17) | 500 | 1.07 | (0.97–1.17) | 92 | **1.30** | **(1.04–1.64)** | 1,262 | 1.01 | (0.94–1.08) | 929 | 0.97 | (0.89–1.05) | 179 | 1.05 | (0.87–1.27) |
|  | ≥160 | 406 | **1.13** | **(1.02–1.25)** | 288 | 1.05 | (0.93–1.19) | 72 | **1.74** | **(1.35–2.24)** | 981 | **1.12** | **(1.03–1.21)** | 685 | 1.02 | (0.93–1.11) | 152 | **1.27** | **(1.04–1.55)** |
| 10–14.9 | <100 | 140 | **1.20** | **(1.02–1.43)** | 108 | 1.20 | (0.99–1.45) | 12 | 1.03 | (0.58–1.83) | 50 | 1.29 | (0.97–1.71) | 36 | 1.21 | (0.87–1.69) | 9 | 1.69 | (0.87–3.28) |
|  | 100–109 | 420 | 1.05 | (0.95–1.17) | 332 | 1.08 | (0.96–1.21) | 45 | 1.10 | (0.80–1.50) | 237 | **1.39** | **(1.22–1.59)** | 174 | **1.34** | **(1.14–1.57)** | 38 | **1.62** | **(1.15–2.28)** |
|  | 110–119 | 1,304 | **1.22** | **(1.14–1.30)** | 1,013 | **1.23** | **(1.14–1.32)** | 135 | 1.20 | (0.98–1.46) | 732 | **1.15** | **(1.06–1.26)** | 540 | **1.11** | **(1.01–1.23)** | 87 | 1.01 | (0.79–1.29) |
|  | 120–129 | 1,552 | **1.16** | **(1.09–1.23)** | 1,168 | **1.13** | **(1.06–1.21)** | 180 | **1.26** | **(1.05–1.50)** | 1,253 | **1.13** | **(1.05–1.21)** | 907 | 1.06 | (0.98–1.16) | 190 | **1.26** | **(1.04–1.52)** |
|  | 130–139 | 1,601 | **1.13** | **(1.06–1.20)** | 1,181 | **1.08** | **(1.01–1.16)** | 211 | **1.36** | **(1.15–1.61)** | 1,779 | **1.14** | **(1.07–1.22)** | 1,293 | 1.08 | (1.00–1.17) | 262 | **1.24** | **(1.04–1.47)** |
|  | 140–149 | 581 | **1.27** | **(1.17–1.39)** | 449 | **1.28** | **(1.16–1.42)** | 78 | **1.55** | **(1.21–1.98)** | 917 | **1.15** | **(1.06–1.24)** | 665 | 1.09 | (0.99–1.19) | 149 | **1.37** | **(1.12–1.68)** |
|  | 150–159 | 243 | 1.10 | (0.96–1.25) | 178 | 1.05 | (0.90–1.22) | 36 | **1.47** | **(1.04–2.07)** | 514 | 1.07 | (0.97–1.18) | 362 | 0.98 | (0.88–1.10) | 86 | **1.31** | **(1.03–1.68)** |
|  | ≥160 | 177 | **1.30** | **(1.12–1.52)** | 139 | **1.34** | **(1.12–1.59)** | 14 | 0.92 | (0.54–1.56) | 430 | **1.23** | **(1.11–1.37)** | 313 | **1.17** | **(1.03–1.32)** | 66 | **1.40** | **(1.07–1.83)** |
| ≥15 | <100 | 23 | 1.21 | (0.80–1.83) | 16 | 1.09 | (0.67–1.78) | 2 | 1.05 | (0.26–4.21) | 14 | **1.90** | **(1.12–3.21)** | 6 | 1.07 | (0.48–2.40) | 2 | 1.93 | (0.48–7.76) |
|  | 100–109 | 85 | **1.42** | **(1.15–1.76)** | 71 | **1.53** | **(1.21–1.94)** | 7 | 1.16 | (0.55–2.44) | 41 | 1.33 | (0.98–1.81) | 33 | 1.40 | (0.99–1.98) | 0 | 0.00 | (–) |
|  | 110–119 | 211 | **1.41** | **(1.22–1.62)** | 167 | **1.44** | **(1.23–1.68)** | 23 | 1.46 | (0.96–2.22) | 131 | **1.28** | **(1.07–1.53)** | 86 | 1.09 | (0.88–1.36) | 19 | 1.37 | (0.86–2.18) |
|  | 120–129 | 252 | **1.26** | **(1.11–1.43)** | 191 | **1.24** | **(1.07–1.43)** | 27 | 1.27 | (0.86–1.87) | 235 | **1.39** | **(1.21–1.59)** | 162 | **1.25** | **(1.06–1.47)** | 32 | 1.38 | (0.96–2.00) |
|  | 130–139 | 287 | **1.29** | **(1.14–1.46)** | 206 | **1.20** | **(1.04–1.38)** | 45 | **1.89** | **(1.39–2.58)** | 362 | **1.45** | **(1.29–1.62)** | 274 | **1.42** | **(1.25–1.62)** | 50 | **1.48** | **(1.09–2.00)** |
|  | 140–149 | 104 | **1.44** | **(1.18–1.75)** | 77 | **1.38** | **(1.10–1.73)** | 12 | 1.56 | (0.88–2.77) | 159 | 1.15 | (0.98–1.35) | 119 | 1.12 | (0.93–1.35) | 26 | 1.38 | (0.92–2.06) |
|  | 150–159 | 52 | **1.52** | **(1.16–2.00)** | 36 | 1.38 | (0.99–1.91) | 9 | **2.37** | **(1.22–4.59)** | 99 | **1.29** | **(1.05–1.58)** | 65 | 1.10 | (0.86–1.42) | 24 | **2.29** | **(1.51–3.48)** |
|  | ≥160 | 41 | **1.63** | **(1.20–2.22)** | 31 | **1.59** | **(1.12–2.27)** | 6 | **2.26** | **(1.01–5.07)** | 89 | **1.43** | **(1.15–1.76)** | 62 | **1.30** | **(1.01–1.67)** | 16 | **1.89** | **(1.14–3.13)** |

BMI, body mass index; CI, confidence interval; CKD, chronic kidney disease; COPD, chronic obstructive pulmonary disease; DM, diabetes mellitus; HR, hazard ratio; IHD, ischemic heart disease; IR, incidence rate (per 1000); KDSQ, Korean Dementia Screening Questionnaire; N, number; SBP, systolic blood pressure; TUG, timed up and go

* Adjusted for sex, income, smoking status, alcohol consumption, exercise, BMI, hemoglobin, KDSQ score, administration of antihypertensive medication, DM, CKD, COPD, IHD, stroke, and depression.

**Supplementary Table S7. Stratified analyses by hypertensive status: hazard ratios and incidence rates of dementia according to diastolic blood pressure when using the group with DBP 70-79 mmHg and TUG result < 10 seconds as a reference.**

|  |  | Without antihypertensive medication | | | | | | | | | With antihypertensive medication | | | | | | | | |
| --- | --- | --- | --- | --- | --- | --- | --- | --- | --- | --- | --- | --- | --- | --- | --- | --- | --- | --- | --- |
|  |  | All dementia | | | Alzheimer’s disease | | | Vascular dementia | | | All dementia | | | Alzheimer’s disease | | | Vascular dementia | | |
| TUG | DBP | N | HR* | (95% CI) | N | HR* | (95% CI) | N | HR* | (95% CI) | N | HR* | (95% CI) | N | HR* | (95% CI) | N | HR* | (95% CI) |
| Total | <60 | 414 | 0.91 | (0.83-1.01) | 314 | 0.91 | (0.81-1.01) | 46 | 0.95 | (0.71-1.28) | 245 | 1.02 | (0.90-1.16) | 166 | 0.95 | (0.81-1.11) | 42 | 1.18 | (0.87-1.62) |
|  | 60-69 | 3,546 | 0.98 | (0.94-1.02) | 2,759 | 1.00 | (0.95-1.04) | 375 | 0.95 | (0.84-1.07) | 2,196 | 1.01 | (0.96-1.06) | 1,634 | 1.03 | (0.97-1.09) | 297 | 0.93 | (0.81-1.06) |
|  | 70-79 | 8,018 | 1.00 | (reference) | 6,103 | 1.00 | (reference) | 894 | 1.00 | (reference) | 6,074 | 1.00 | (reference) | 4,445 | 1.00 | (reference) | 886 | 1.00 | (reference) |
|  | 80-89 | 7,658 | **1.03** | **(1.00-1.06)** | 5,799 | 1.03 | (0.99-1.07) | 946 | **1.11** | **(1.01-1.22)** | 7,868 | **1.06** | **(1.02-1.09)** | 5,683 | **1.04** | **(1.00-1.09)** | 1216 | **1.12** | **(1.03-1.22)** |
|  | 90-99 | 1,843 | **1.08** | **(1.03-1.14)** | 1,365 | **1.06** | **(1.00-1.13)** | 264 | **1.33** | **(1.16-1.53)** | 3,059 | **1.11** | **(1.06-1.15)** | 2,218 | **1.10** | **(1.04-1.15)** | 454 | **1.12** | **(1.00-1.26)** |
|  | 100-109 | 411 | 1.08 | (0.98-1.19) | 311 | 1.08 | (0.97-1.22) | 53 | 1.17 | (0.89-1.54) | 892 | **1.20** | **(1.12-1.29)** | 650 | **1.19** | **(1.10-1.30)** | 133 | **1.23** | **(1.02-1.47)** |
|  | ≥110 | 76 | **1.25** | **(1.00-1.57)** | 53 | 1.16 | (0.89-1.52) | 13 | **1.81** | **(1.05-3.14)** | 176 | **1.24** | **(1.07-1.44)** | 112 | 1.08 | (0.90-1.31) | 36 | **1.70** | **(1.22-2.37)** |
| <10 | <60 | 251 | **0.83** | **(0.73-0.95)** | 189 | **0.82** | **(0.71-0.94)** | 26 | 0.81 | (0.55-1.20) | 153 | 1.00 | (0.85-1.18) | 102 | 0.91 | (0.75-1.11) | 26 | 1.13 | (0.77-1.68) |
|  | 60-69 | 2,493 | 1.01 | (0.96-1.06) | 1,936 | 1.02 | (0.97-1.08) | 258 | 0.95 | (0.82-1.10) | 1,482 | 1.02 | (0.96-1.09) | 1,106 | 1.04 | (0.97-1.11) | 200 | 0.93 | (0.79-1.09) |
|  | 70-79 | 5,495 | 1.00 | (reference) | 4,195 | 1.00 | (reference) | 616 | 1.00 | (reference) | 4,049 | 1.00 | (reference) | 2,991 | 1.00 | (reference) | 598 | 1.00 | (reference) |
|  | 80-89 | 5,133 | **1.04** | **(1.00-1.08)** | 3,890 | 1.04 | (0.99-1.08) | 621 | 1.09 | (0.98-1.22) | 5,066 | **1.05** | **(1.01-1.09)** | 3,647 | 1.02 | (0.97-1.07) | 790 | 1.11 | (0.99-1.23) |
|  | 90-99 | 1,195 | **1.07** | **(1.00-1.14)** | 891 | 1.05 | (0.98-1.13) | 181 | **1.39** | **(1.18-1.64)** | 2,014 | **1.13** | **(1.07-1.19)** | 1,466 | **1.11** | **(1.04-1.18)** | 287 | 1.09 | (0.95-1.25) |
|  | 100-109 | 275 | 1.12 | (0.99-1.26) | 205 | 1.11 | (0.96-1.27) | 37 | 1.26 | (0.90-1.75) | 587 | **1.23** | **(1.13-1.35)** | 425 | **1.21** | **(1.09-1.35)** | 86 | 1.22 | (0.97-1.53) |
|  | ≥110 | 51 | **1.33** | **(1.01-1.75)** | 35 | 1.21 | (0.87-1.69) | 10 | **2.19** | **(1.17-4.08)** | 117 | **1.27** | **(1.05-1.52)** | 74 | 1.09 | (0.87-1.37) | 21 | 1.49 | (0.96-2.30) |
| 10-14.9 | <60 | 139 | **1.26** | **(1.07-1.49)** | 111 | **1.31** | **(1.08-1.58)** | 16 | 1.38 | (0.84-2.26) | 76 | 1.22 | (0.97-1.53) | 54 | 1.18 | (0.90-1.54) | 14 | 1.52 | (0.90-2.59) |
|  | 60-69 | 900 | **1.10** | **(1.03-1.18)** | 703 | **1.12** | **(1.03-1.21)** | 100 | 1.14 | (0.92-1.41) | 601 | **1.18** | **(1.08-1.29)** | 454 | **1.21** | **(1.20-1.34)** | 81 | 1.07 | (0.85-1.35) |
|  | 70-79 | 2,156 | **1.19** | **(1.14-1.25)** | 1,629 | **1.17** | **(1.11-1.24)** | 230 | **1.16** | **(1.00-1.36)** | 1,712 | **1.19** | **(1.12-1.26)** | 1,235 | **1.16** | **(1.09-1.24)** | 242 | 1.14 | (0.98-1.33) |
|  | 80-89 | 2,162 | **1.20** | **(1.14-1.26)** | 1,635 | **1.18** | **(1.12-1.25)** | 285 | **1.40** | **(1.22-1.61)** | 2,338 | **1.25** | **(1.19-1.32)** | 1,684 | **1.22** | **(1.15-1.29)** | 368 | **1.34** | **(1.18-1.53)** |
|  | 90-99 | 530 | **1.26** | **(1.15-1.37)** | 386 | **1.20** | **(1.08-1.33)** | 66 | **1.36** | **(1.06-1.76)** | 873 | **1.25** | **(1.16-1.34)** | 637 | **1.23** | **(1.13-1.34)** | 131 | **1.27** | **(1.05-1.54)** |
|  | 100-109 | 112 | 1.17 | (0.97-1.41) | 88 | 1.21 | (0.98-1.50) | 13 | 1.16 | (0.67-2.01) | 261 | **1.37** | **(1.21-1.55)** | 192 | **1.36** | **(1.17-1.57)** | 38 | 1.36 | (0.98-1.89) |
|  | ≥110 | 19 | 1.15 | (0.73-1.80) | 16 | 1.28 | (0.78-2.09) | 1 | 0.52 | (0.07-3.68) | 51 | **1.47** | **(1.12-1.94)** | 34 | 1.33 | (0.95-1.86) | 13 | **2.54** | **(1.46-4.40)** |
| ≥15 | <60 | 24 | **1.54** | **(1.03-2.29)** | 14 | 1.16 | (0.69-1.96) | 4 | 2.41 | (0.90-6.45) | 16 | 1.55 | (0.95-2.53) | 10 | 1.32 | (0.71-2.45) | 2 | 1.30 | (0.32-5.22) |
|  | 60-69 | 153 | **1.26** | **(1.08-1.48)** | 120 | **1.28** | **(1.07-1.54)** | 17 | 1.34 | (0.83-2.17) | 113 | **1.29** | **(1.07-1.55)** | 74 | 1.14 | (0.90-1.43) | 16 | 1.24 | (0.75-2.04) |
|  | 70-79 | 367 | **1.39** | **(1.25-1.55)** | 279 | **1.38** | **(1.22-1.56)** | 48 | **1.69** | **(1.26-2.27)** | 313 | **1.39** | **(1.24-1.56)** | 219 | **1.32** | **(1.15-1.51)** | 46 | **1.40** | **(1.04-1.89)** |
|  | 80-89 | 363 | **1.29** | **(1.16-1.44)** | 274 | **1.28** | **(1.13-1.44)** | 40 | 1.28 | (0.93-1.76) | 464 | **1.54** | **(1.40-1.70)** | 352 | **1.58** | **(1.42-1.77)** | 58 | **1.31** | **(1.00-1.71)** |
|  | 90-99 | 118 | **1.70** | **(1.42-2.05)** | 88 | **1.66** | **(1.34-2.05)** | 17 | **2.21** | **(1.36-3.58)** | 172 | **1.43** | **(1.22-1.66)** | 115 | **1.29** | **(1.07-1.55)** | 36 | **2.02** | **(1.44-2.82)** |
|  | 100-109 | 24 | 1.29 | (0.86-1.92) | 18 | 1.26 | (0.79-2.00) | 3 | 1.45 | (0.47-4.51) | 44 | **1.36** | **(1.01-1.83)** | 33 | 1.38 | (0.98-1.94) | 9 | 1.89 | (0.98-3.66) |
|  | ≥110 | 6 | **2.58** | **(1.16-5.75)** | 2 | 1.12 | (0.28-4.48) | 2 | **8.06** | **(2.01-32.3)** | 8 | 1.19 | (0.59-2.38) | 4 | 0.80 | (0.30-2.14) | 2 | 2.05 | (0.51-8.22) |

BMI, body mass index; CI, confidence interval; CKD, chronic kidney disease; COPD, chronic obstructive pulmonary disease; DBP, diastolic blood pressure (mmHg); DM, diabetes mellitus; HR, hazard ratio; IHD, ischemic heart disease; IR, incidence rate (per 1000); KDSQ, Korean Dementia Screening Questionnaire; N, number; TUG, timed up and go (seconds)

* Adjusted for sex, income, smoking status, alcohol consumption, exercise, BMI, hemoglobin, KDSQ score, administration of antihypertensive medication, DM, CKD, COPD, IHD, stroke, and depression.
